# Superparamagnetic state in La_0.7_Sr_0.3_MnO_3_ thin films obtained by rf-sputtering

**DOI:** 10.1038/s41598-020-59334-3

**Published:** 2020-02-13

**Authors:** M. C. Ramírez Camacho, C. F. Sánchez Valdés, M. Curiel, J. L. Sánchez Llamazares, J. M. Siqueiros, O. Raymond Herrera

**Affiliations:** 1 Centro de Nanociencias y Nanotecnología, Universidad Nacional Autónoma de México, AP 14, 22860 Ensenada, Baja California, México; 2 Instituto de Ingeniería, Universidad Autónoma de Baja California, Mexicali, Blvd. Benito Juarez s/n, 21280 Baja California, México; 3 División Multidisciplinaria, Ciudad Universitaria, Universidad Autónoma de Ciudad Juárez, J. J. Macías Delgado # 18100, Ciudad Juárez, 32579 Chihuahua, México; 4 Instituto Potosino de Investigación Científica y Tecnológica A.C., Camino a la Presa San José 2055, Col. Lomas 4ª sección, San Luis Potosí, 78216 México

**Keywords:** Materials science, Nanoscience and technology, Physics

## Abstract

A novel superparamagnetic state has been observed in high quality La_0.7_Sr_0.3_MnO_3_ (LSMO) thin films directly grown by rf-sputtering on SiO_x_/Si(100) substrates. The films are nanostructured without grain boundaries, constituted by locally epitaxial nanoregions grown layer-by-layer with *out-of-plane* (012) preferential orientation, induced by the constrain of the native silicon oxide. Low magnetic field ZFC-FC magnetization curves show a cross-over from superparamagnetic to ferromagnetic state dependent of the thickness. The thicker film (140 nm) exhibits typical ferromagnetic order. The thinner films (40 and 60 nm) exhibit superparamagnetic behavior attributed to interacting ferromagnetic monodomain nanoregions with critical size, random *in-plane* oriented, where the inter-monodomain boundaries with surface spin-glass structure regulate the blocking of magnetization depending on the magnetic field intensity. M(H) hysteresis loops showed noticeable coercive fields in all samples, larger than those reported for LSMO. Such properties of half-metal LSMO film foresee potential integration in new Si-technology nanodevices in Spintronics.

## Introduction

Perovskite mixed valence manganites have attracted great interest since the discovery of the colossal magnetoresistance (CMR) effect^1^. Film growth of oxides R_1−x_B_x_MnO_3_, where R and B are rare-earth and alkaline metals, respectively, is particularly interesting. These oxide materials are desirable due to their chemical flexibility, which allows the study of the structural, electronic and magnetic properties and possible couplings between them that enables their use in Spintronics and novel functional electronic devices such as magnetic random access memory, disk-driven reads, magnetic field sensors and infrared detectors, among others^1,2^.

In particular, the lanthanum strontium manganite La_1−x_Sr_x_MnO_3_ is one of the most promising materials for devices operating at room temperature (RT); when La_1−x_Sr_x_MnO_3_ is doped with the optimum Sr concentration of x = 0.3 (LSMO), the manganite has a Curie temperature (T_C_) of ∼370 K^3^. This manganite is characterized by a colossal magnetoresistance at RT range associated to the ferromagnetic ordering of manganese magnetic moments^4^, to high magnetic anisotropy and a high spin polarization (nearly 100%) as predicted by first-principles calculations^1,3^. Usually, LSMO thin films are used as buffer layers in many device structures such as magnetic field sensing, resistive sensor and magnetic storage applications at RT^1,5^. Moreover, differing previous studies on ultrathin (110) oriented LSMO films grown on SrTiO_3_ (110) substrates have reported a room temperature insulating magnetic phase depending on thickness, induced by strain, but with ferromagnetic structure with T_C_ ∼330 K for thickness above 15 unit cells^6^ or a canted antiferromagnetic structure with higher T_C_ ∼560 K for thickness below the critical value of 10 unit cells^7^. On the other hand, direct silicon-based heterostructures greatly facilitate the fabrication of microelectronic devices. A plethora of reports have documented the high reactivity of silicon with oxygen which, in turn promotes the disruption of epitaxy due to the presence of an amorphous SiO_x_ native layer on the Si substrates^8^. Therefore it has been common practice to grow epitaxial buffer layers of CaTiO_3_, NdGaO_3_, DyScO_3_ or Sr_2_AlTaO_6_ on different monocrystalline substrates, particularily on SrTiO_3_ and LaAlO_3_ substrates^4^.

Based on the nature of the deposition process, the methods employed for thin oxide films can be divided into two groups i.e. physical and chemical methods. Most used physical deposition processes include vacuum evaporation, pulsed laser deposition, molecular beam epitaxy (MBE), and sputtering^1^. Zheng *et al*.^9^ obtained LSMO epitaxial thin films grown on ferroelectric 0.68Pb(Mg_1∕3_Nb_2∕3_)O_3_–0.32PbTiO_3_ (PMN-PT) single-crystal substrates by magnetron sputtering and found that the resistivity of the LSMO films could be tuned by electric fields. Zhang *et al*.^10^ provided an alternative approach by growing highly oriented ferromagnetic LSMO films on PMN-PT using a physicochemical sol-gel technique. They obtained magnetic hysteresis loops with coercive field H_C_ ~50 Oe at RT and a typical ferromagnetic (FM) behavior at 10 K with H_C_ ~300 Oe. Other works that stand out are those of Gu *et al*.^11^ and Yu *et al*.^12^, focused in ferromagnetic and structural properties and novel magnetic phenomena; however, those works reported low coercive fields even at low temperatures. Studies performed by Martin *et al*.^13^ and Gajek *et al*.^14^ reported comparable values of H_C_ = 40 Oe at 10 K for La_0.7_Sr_0.3_MnO_3_^15^ and H_C_ = 310 Oe at 10 K for La_0.1_Bi_0.9_MnO_3_^14^, respectively.

Additionally, the physical properties of superparamagnetic (SPM) nanoparticles of La_0.7_Sr_0.3_MnO_3_ have been reported as early as 2006^16^. However, such nanoparticles are still multi-domain, and their magnetic dynamics naturally differs from that of a superparamagnetic system. In the literature there seems to be no further studies in superparamagnetic LSMO thin films. As far as we know, we believe that no other authors have published on this matter besides the early efforts made by Krivoruchko *et al*.^15,16^ on superparamagnetism studies on ~12 nm LSMO single domain nanoparticles.

Thus, the aim of this work is to study the structural and magnetic properties of LSMO thin films of different thickness grown on SiO_x_/Si substrates by rf sputtering. The observed local epitaxy, high crystalline orientation, the SPM behavior at lower applied magnetic fields and the high coercive field FM behavior at higher applied magnetic fields are presented and discussed.

## Experimental Details

A stoichiometric LSMO ceramic target was fabricated by typical solid-state reaction using high purity La_2_O_3_, SrCO_3_ and SrCO_3_ precursor powders. LSMO thin films were directly grown on commercial SiO_x_/Si (SOS) substrates with (100) orientation, using rf-magnetron sputtering technique. Optimal deposit parameters for LSMO films were: 2.0 × 10^–5^ Torr base pressure, 5 cm target-substrate distance, 773 K substrate temperature, 250 rf power, partial pressures of Ar and O totaling 20 mTorr. The deposition times were set to 15, 30 and 60 min which lead to thickness values of 40, 60 and 140 nm, respectively, measured by transmission electron microscopy (TEM) (see below). Thus the sample label was chosen as L40, L60 and L140 according to the thickness values.

X-ray diffraction patterns were collected with an X-Pert PRO MRD diffractometer from PANANALYTICAL, using Cu-Kα_1_ radiation with wavelength of 1.540598 Å. Cross-sectional specimens were prepared using the focused ion beam (FIB) technique, available in a JEOL JIB-4500 scanning electron microscope; before, all LSMO/SOS stacks were coated with a gold film to protect from Ga beam damage. High-angle annular dark-field images and local chemical composition characterization was realized by scanning transmission electron microscopy (STEM) using a JEOL JEM-2100F microscope equipped with energy dispersive spectroscopy (EDS). Local structural analysis was realized by high resolution TEM technique using a JEOL JEM-2200FS microscope employing an accelatring voltage of 200 kV. The *Diamond* software (version 4)^17^ was used for the structural simulation and analysis.

A DynaCool 9 T platform from QUANTUM DESIGN, equipped with vibrating sample magnetometry technique, was used for magnetic characterization. Magnetization curves as functions of temperature M(T), were measured in the 2.5 K to 400 K temperature range under an applied magnetic field H = 200 Oe. The M(T) curves, in zero-field-cooled (ZFC) and field-cooled (FC) regime, were obtained in no-over-shoot mode with a heating and cooling rate of 1 K/min. High magnetic field FC curves obtained under 50 kOe were measured from 400 K to 2.5 K. Magnetization curves as functions of the applied magnetic field M(H) were measured, at selected temperatures from 2.5 K to 400 K, between – 40 kOe and + 40 kOe using a step of 100 Oe/s. The M(H) curves were normalized to the total volume of the LSMO layer for each sample, using the thickness values obtained by TEM. For all M(H) curves, the diamagnetic contribution of the SiO_x_/Si substrate was subtracted.

## Results and Discussion

### Structural characterization

**Figure 1:**
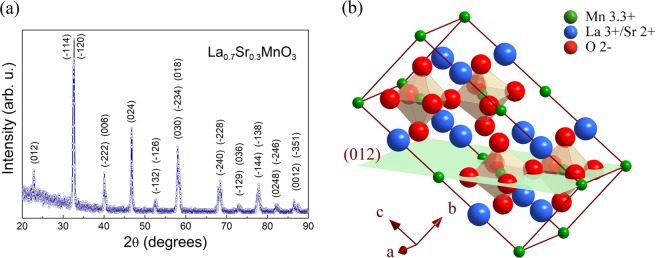
(**a**) XRD pattern of stoichiometric La_0.7_Sr_0.3_MnO_3_ (LSMO) ceramic target. It is indexed with the rhombohedral $R\bar{3}c$ space group (167). (**b**) Hexagonal unit cell of LSMO; the highlighted plane corresponds to the (012) plane.

Figure 1a shows the X-ray diffraction data of the stoichiometric La_0.7_Sr_0.3_MnO_3_ ceramic target, which exhibits the standard polycrystalline pattern. The pattern can be indexed with the rhombohedral crystalline structure ($R\bar{3}c$ space group, SG 167) with hexagonal lattice parameters of *a*_LSMO_ = 5.5213 Å and *c*_LSMO_ = 13.4130 Å, in good correspondence with the PDF-01–089–8095 file reported for LSMO^18^. The obtained parameters agree with those of *a* = 5.52 Å and *c* = 13.36 Å reported by Choi *et al*.^19^ and according with the structural phase diagram of the La_1-x_Sr_x_MnO_3_^20^. Figure 1b illustrates the hexagonal cell of LSMO corresponding to $R\bar{3}c$ symmetry.

**Figure 2:**
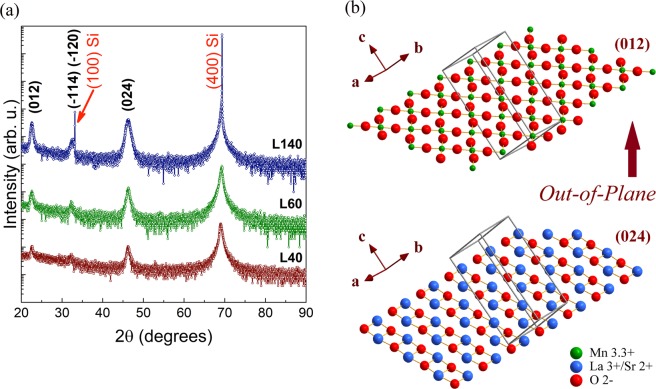
(**a**) XRD patterns of the La_0.7_Sr_0.3_MnO_3_ thin films L40, L60 and L140 grown on SiO_2_/Si(100) substrates with 40 nm, 60 nm and 140 nm thickness, respectively. (**b**) Structural simulation of an LSMO film using the parameters obtained from XRD data, illustrating the in-plane atomic array on the (012) and (024) planes which are perpendicular to the out-of-plane direction.

XRD patterns of LSMO thin films grown on SOS substrates with different thickness are shown in Fig. 2a. The peaks observed at 2θ = 69.05° and 2θ = 32.50° correspond to the (400) and (100) planes of the Si substrate. All LSMO/SOS samples show highly textured growth of LSMO thin films with the (012) *out-of-plane* preferential orientation described by the indexed peaks (012) positioned at 2θ ∼22.58° and (024) at 2θ ∼46.25° corresponding to those planes which have the composition and atoms arrangement illustrated in Fig. 2b. Such indexing corresponds to the rhombohedral structure ($R\bar{3}c$ space group, *SG 167*) in agreement with the structural description above on the LSMO target. The Table 1 shows the full with at half maximum (FWHM) values calculated from the (024) peaks. The widening of the (024) peak and the presence of the peak at 2θ = 32.36° corresponding to $(\bar{1}20)$ and $(\bar{1}14)$ planes, denotes the degree of *out-of-plane* misorientation of the film. Such misorientation grows as thicknesss increases as corroborated by the increase of both the (024) peak widening and the relative intensity of the peak at 2θ = 32.36°. Moreover, grazing-incidence XRD measurements (illustrated in Supplementary Fig. S1) were performed on L60 sample by means of omega-2theta (*ω*−2θ) scan and *ω*-scan (rocking curve). The *ω*−2θ pattern obtained at optimal *ω* = 3° confirms the highly (012) *out-of-plane* preferential orientation, while the *ω*-scan around to *ω* = 3° at 2θ = 22.45° corresponding to the (012) maximum that exhibits a peak with a FWHM = 3°, demonstrates the *out-of-plane* misorientation which can be associated with a nanostructured growth. As will be discussed below, these growth features can be related with an *in-plane* ramdom orientation of nanodomains imposed by the non-crystalline surface of the native amorphous silicon oxide film on the Si substrate. Pole figures (*ψ*,*Φ*- scan) measured at 2θ values corresponding to other planes different to those of the *out-of-plane* orientation as $(\bar{1}14)$/$(\bar{1}20)$ at 2θ = 32.36° and (030) at 2θ = 58.17° exhibit constant intensity profiles, evincing the *in-plane* ramdom orientation of nanodomains (see the Supplementary Fig. S2).

**Table 1:** Values of the FWHM calculated from the (024) peaks, the hexagonal lattice parameters and thickness obtained for LSMO thin films.

	Sample		
	L40	L60	L140
Thickness (nm)	40	60	140
*a*_H_ (Å)	5.52	5.53	5.52
*c*_H_ (Å)	13.77	13.70	13.78
FWHM (°)	0.71	0.75	1.00

Using the structural and XRD simulation and the XRD data, the hexagonal lattice parameters for each sample were obtained and are reported in Table 1. These parameters are in good correspondence with those reported by Hibble *et al*.^18^ (ICSD 88409) of *a = *5.5085 Å and *c = *13.717 Å at RT for La_0.7_Sr_0.3_MnO_3_. The lattice parameters calculated here for the ceramic target and LSMO films are taken as evidence that the stoichiometric and chemical composition of the La_0.7_Sr_0.3_MnO_3_ compound is fulfilled.

As can be noted, our results show that all samples exhibit good crystalline features, regarding crystalline structure and the preferential orientation of growth, independently of the change in thickness. These features distinguish our procedure from other studies of LSMO thin films with thickness of 350 nm, grown by sputtering on SOS substrates where the crystalline quality and the growth orientation are influenced by the rf-sputtering conditions and oxygen pressure^21,22^.

**Figure 3:**
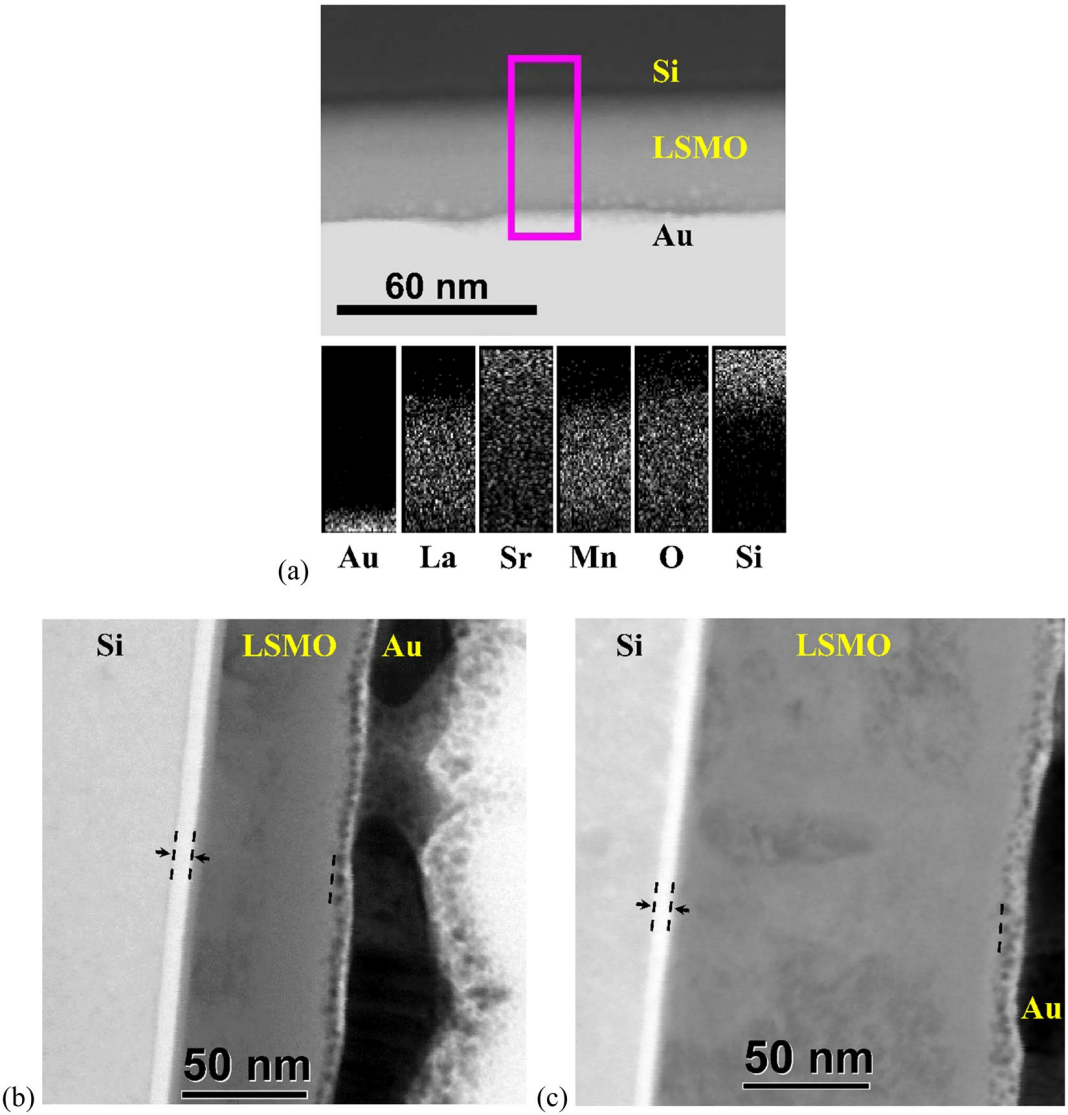
Cross-section HAADF STEM analysis of LSMO/SOS stacks. (**a**) EDS results on the 40 nm (L40) thick LSMO thin film (the Au layer is a protective layer for FIB preparation). Representative images of LSMO films with thickness of (**b**) 60 nm (L60) and (**c**) 140 nm (L140), respectively. The dash lines in (**b**) and (**c**) indicate the silicon oxide layers.

Figure 3 illustrates representative cross-section images of LSMO/SOS stacks obtained by high-angle annular dark-field (HAADF) imaging method from STEM. It demonstrates that all LSMO films growth on SOS substrates are highly homogenous, without demarcated grain boundaries, and allow to obtain good valuation of the average thickness values reported in Table 1. Figure 3a shows the chemical mapping obtained from EDS analysis for the L40 sample in the area indicated with the rectangle. All expected elements are present. The Au signal comes from the layer for the cross-sectional sample preparation. The Au, La, Sr, Mn, O and Si signals correspond to the M_α1_, L_α1_, L_α1_, K_α1_, K_α1_ and K_α1_ transitions, respectively. As can be seen, a high signal is observed on the Si zone of the Sr rectangle. This effect can be explained by the strong emission coming from the monocrystalline Si due to overlapping of the Sr-L_α1_ signal (1.807 keV for high level) with those of the Si- K_α1_ (1.740 keV for high level).

**Figure 4:**
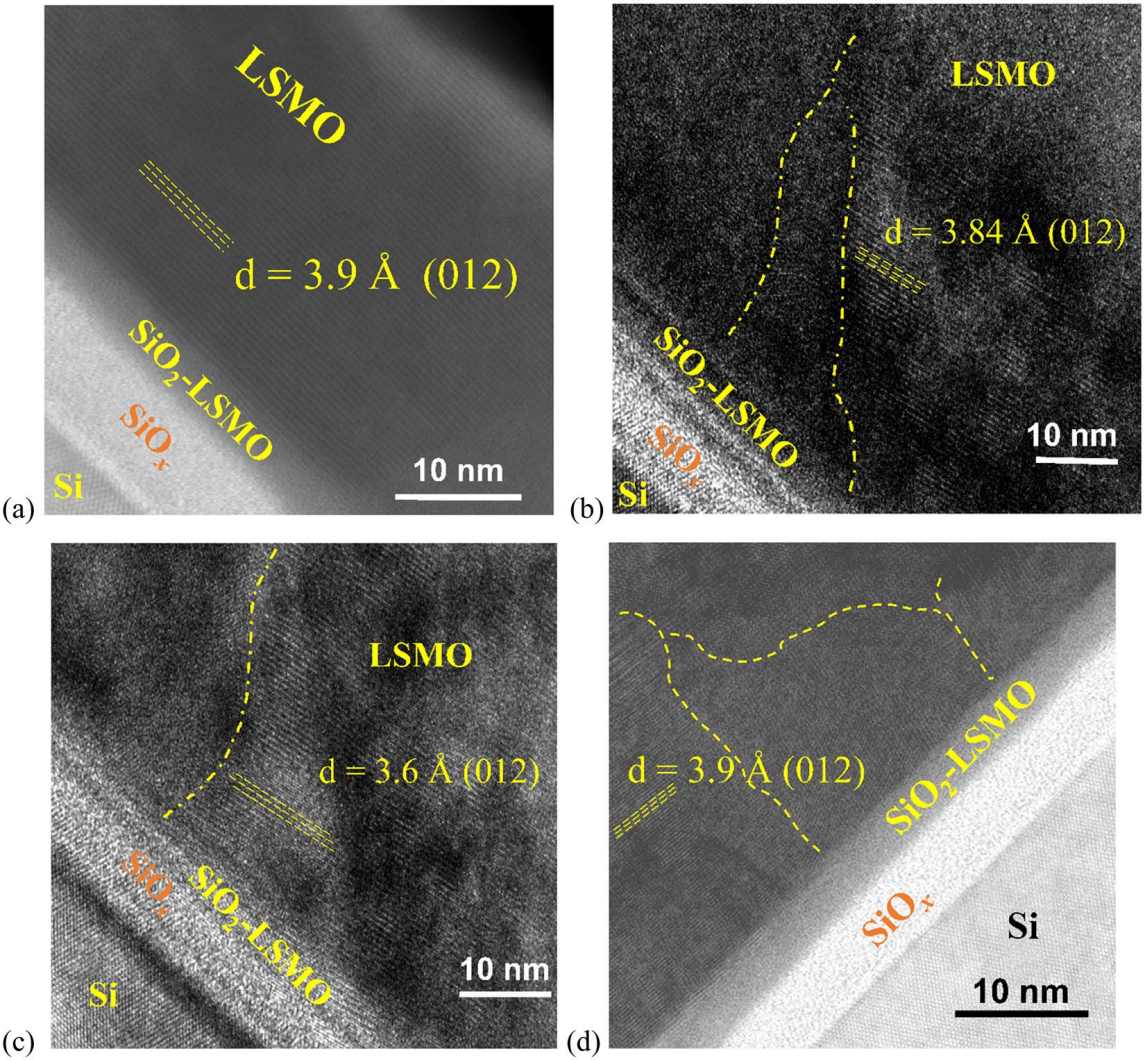
Cross-section high resolution TEM micrographs of the sputtered LSMO films with thickness of (**a**) 40 nm (L40), (**b**) 60 nm (L60), and (**c**) and (**d**) 140 nm (L140). Dash lines are guides for the eyes.

In addition, representative cross-section high-resolution TEM micrographs of all LSMO/SOS samples are illustrated in Fig. 4. The LSMO thin films exhibit excellent crystalline growth on the native silicon oxide layer with thickness ~ 6 nm. In Fig. 4, it is possible to observe that all LSMO films have the same preferential growth orientation. The average value of 3.81 Å, obtained from the measured interplanar distances, is in good correspondence with the expected value of 3.8933 Å associated to (012) plane (Fig. 1b) in the standard XRD pattern, which confirms the (012) preferential orientation.

In our work, the LSMO thin film grew relaxed without appreciable grain boundaries, and a continuous connection between the LSMO film with the silicon oxide layer with an LSMO-SiO_x_ interface thickness of ~ 4 nm (Fig. 4) suggesting a local epitaxial growth of LSMO. All these features establish a noticeable distinction with the sharp grain boundaries observed in the compressive strained 50 nm LSMO thin film grown by molecular beam epitaxy (MBE) on epitaxial CaTiO_3_ buffered silicon substrates and the formation of a 10 nm amorphous SiO_x_ layer reported by Adamo *et al*.^5^

To understand such unusual epitaxial coupling between the LSMO films here obtained and the native silicon oxide of the Si wafers, structural simulation can be used. Figures 1b and 2b show that on the LSMO structure, the (012) and (024) planes are described by crossed Mn–O–Mn and La/Sr–O–La/Sr chains, respectively; where, according to $R\bar{3}c$ symmetry, the Mn^3+^/Mn^4+^ ions that are occupying the octahedral *B-*site are six-fold coordinated, while the ions La^3+^/Sr^4+^ occupying *A-*site are nine-fold coordinated. Moreover, according to Fig. 2b, the LSMO growth with (012) *out-of-plane* orientation could be consider as a layer-by-layer growth of the (012) and (024) planes as is observed by HRTEM in Fig. 4. Thus, such a highly textured (012) growth of the LSMO thin films and specially the increase of the *a*_H_ lattice parameter respect to those reported for LSMO, could be a result of the local incommensurate coupling of a crossed Mn–O–Mn chains arrangement with the two-dimensional Si–O–Si array at the surface of the native amorphous ultrathin silicon oxide film on the Si wafers. Previous theoretical studies using reactive molecular dynamics^8,23^ have demonstrated that the upper layers of the native silicon oxide have the SiO_2_ stoichiometry and the planar rings with lowest energy formation are four-membered rings^23^. Consequently, the Mn–O–Mn arrangement of (012) planes conforming four-membered rings (Fig. 2b), and characterized by a Mn–O bond length of 1.94 Å, O–Mn–O angles of 89.96° and 90.04°, and Mn–O–Mn angle of 166.50°, can be constrained by the local ordering parameters of the Si–O–Si surface characterized by a Si–O bond length of 1.61 Å, O–Si–O angle of ~110°, and Si–O–Si angle of 160° as reported by previous theoretical studies^8,23^. Such coupling between the Mn-O and Si-O four-membered rings could be the mechanism that explain the strain relaxation close to the LSMO/SiO_2_ interface and the local epitaxial growth of highly (012) *out-of-plane* oriented naonoregions, random *in-plane* oriented, which are very difficult to be observed by TEM due to the amorphous nature of the silicon oxide layer. This coupling at the LSMO/SiO2 interface release the strain and stress interactions inhibiting the grain boundaries formation.

### Magnetic characterization

**Figure 5:**
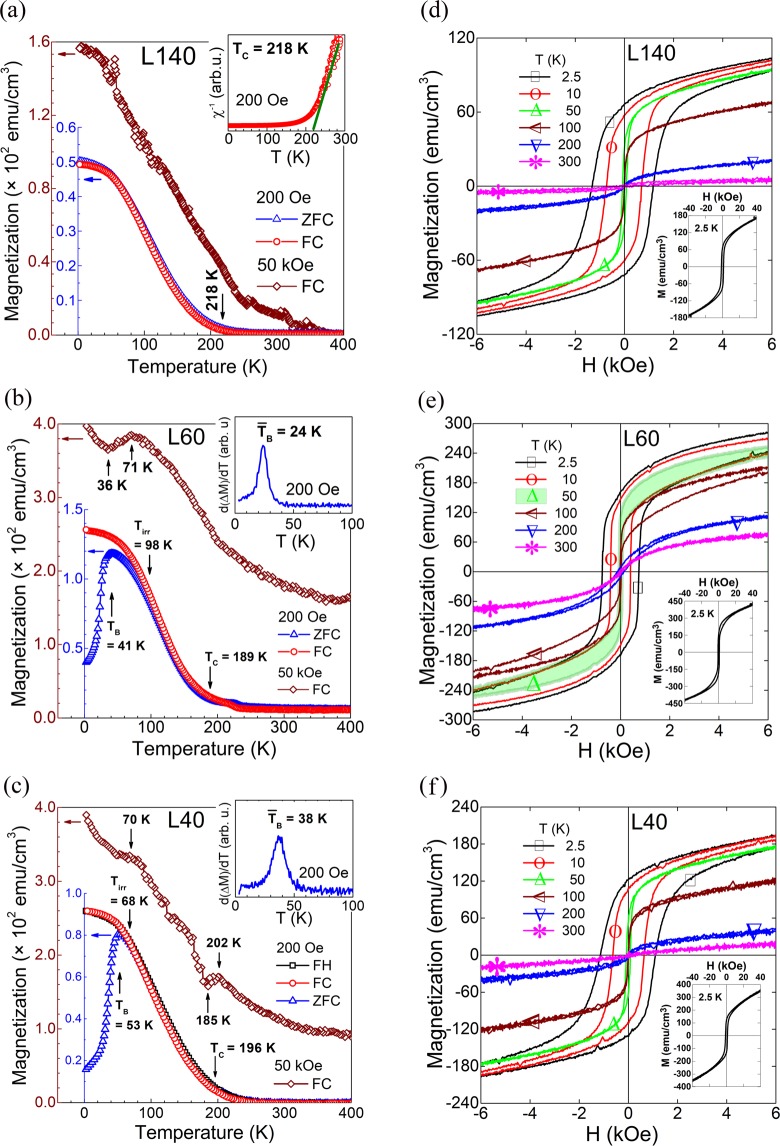
Magnetic characterization of the LSMO/SOS stacks. Temperature dependence of the magnetization at low (200 Oe) and high (50 kOe) applied magnetic fields of (**a**) L140, (**b**) L60, and (**c**) L40 samples. The insets are the (**a**) reciprocal susceptibility (χ^−1^) plot, and (**b**) and (**c**) are the derivate d(ΔM)/dT vs T plots, with ΔM = M_ZFC_-M_FC_. M(**H**) hysteresis loops measured at different temperatures with H_MAX_ = 40 kOe for (**d**) L140, (**e**) L60, and (**f**) L40. Inside all figures, the complete M(H) loop measured at 2.5 K.

Figures 5a,c show the *in-plane* M(T) behaviors under a magnetic field of 200 Oe, measured from 2.5 K to 400 K, of the LSMO/SOS samples with different thicknesses of the LSMO thin films, and they were not corrected due to the very small magnetic contribution of the SiO_*x*_/Si(100) substrate around of 6 × 10^−6^ emu at 200 Oe. L140 exhibits a typical ferromagnetic behavior (Fig. 5a) as the ZFC and FC curves follow the same path, characterized by the Curie temperature T_C  _ = 218 K determined from the reciprocal susceptibility (*χ*^−1^) plott using the ZFC magnetization data as illustrated in the inset in Fig. 5a. Previous studies on LSMO films grown on dissimilar substrates have reported ZFC curves exhibiting a FM behavior with Curie temperatures between 330 K to 560 K for thin films^5–7,24^ and 370 K for bulk samples^25^; but recently, studies on LSMO:CuO nanocomposite films have reported T_C_ values around 220 K and 250 K^26^. The lower value of 218 K here obtained could be correlated with the *in-plane* misorientation and the inter-nanoregions disordered phase boundaries with random *in-plane* orientations inherited from the two-dimensional Si–O–Si array at the surface of the native amorphous where the LSMO thin film grew.

The thinner films L40 and L60 exhibit strong splitting between the ZFC and FC magnetization curves as can be seen in Fig. 5b,c, respectively, where the ZFC curves exhibit a magnetization maximum followed by a magnetization descent when temperature decreases down to 2.5 K. Moreover, it can be observed that the split occurs at higher temperatures than those corresponding to maximum values of magnetization in ZFC curves. Such behavior of the ZFC curves for L40 and L60 samples correspond to a typical superparamagnetic (SPM) behavior described by four characteristic temperatures: i. the paramagnetic-SPM transition temperature T_C_, ii. the blocking temperature T_B_ corresponding to maximum value of ZFC data which is proportionally correlated to the critical particle size^16^, iii. the irreversibility temperature T_irr_ at which ZFC magnetization departs from that of FC, and iv. the temperature of the maximum of the derivative d(M_FC_-M_ZFC_)/dT vs. T curve, known as the mean blocking temperature ${\bar{{\rm{T}}}}_{{\rm{B}}}$^27^. The SPM behavior for the LSMO systems has been reported in studies on 12 nm single-domain nanoparticles obtained by joint deposition^16^ or 20 nm multidomain nanoparticles prepared via sol-gel^28^; but, as far as it has been possible to review, such behavior in LSMO thin films have only been reported in our own previous report^29^.

The observed SPM state on the nanostructured LSMO layers can be associated to interacting ferromagnetic monodomain nanoregions, whose average size should be smaller than a certain critical size as a condition to transit from a macroscopic ferromagnetic interaction to a macroscopic paramagnetic behavior^27–32^. These nanoregions, delimited with dashed lines in Fig 4b,d, are induced by the local constrain of the SiO_2_ surface as was discussed above, but without appreciable grain boundaries for both samples. It is known that the SPM nanoparticles have a characteristic surface layer with spin-glass structure, in correspondence with the larger surface-to-volume ratio, which produces an anisotropic field that frustrates the inner magnetic moments^30,31^. Thus, the SPM behavior here observed at low magnetic field, is attributed to the antiparallel coupling between the surface spin-glass layers at both sides of the nanoregions interface, that shield the inner FM magnetic moments and weakens the inter-monodomain exchange. Such magnetic energy barrier, associated with the surface anisotropic field at the nanoregions boundaries, can be overcome with the increase of the external magnetic field intensity. With this, in ZFC curves, for T < T_B_ the magnetization of the blocked monodomains is oriented antiparallel with each other so that the free energy is minimized causing the total magnetization to drop sharply with the temperature decrease. When T > T_B_, thermal vibrations promote the unblocking of monodomains which will no longer contribute to the raise of the total magnetization because their magnetizations are randomly oriented. For T > T_C_ the monodomain nanoregions lose their spontaneous magnetization, each atomic magnetic moment is randomly oriented, and the system behaves as a classic paramagnetic material.

From the ZFC curves the values of T_B_ = 41 K, T_irr_ = 98 K and ${\bar{{\rm{T}}}}_{{\rm{B}}}$ = 24 K were obtained for L60 (Fig. 5b) and of T_B_ = 53 K, T_irr_ = 68 K and ${\bar{{\rm{T}}}}_{{\rm{B}}}$ = 38 K for L40 (Fig. 5c). Meanwhile, the T_C_ values for the SPM L60 (T_C_ = 189 K) and L40 (T_C_ = 196 K) samples were determined from the reciprocal susceptibility (*χ*^−1^) plott according to the Curie-Wess law using the FC magnetization curve (see the Supplementary Fig. S3). In comparison, it can be seen that the L60 sample has a wider particle size distribution as its T_irr_ value is higher and its T_B_ value is lower respect to those of the L40 film and thus, since T_B_ is proportional to the critical particle size, the size of monodomains in L60 film can be assumed smaller than those for the L40 film which is thinner. Therefore, for L60 there is a higher fraction of unblocked nanoregions at temperatures above T_B_ and below T_irr_ that do not contribute to the increase of magnetization in the ZFC curve but can contribute in the FC curve. Meanwhile, for L40 such fraction of unblocked nanoregions is smaller as T_irr_ is closer to T_B_ and the size distribution is more narrow, i.e. the *in-plane* monodomain size is more homogeneous. However, for L40 the derivate d(M_FC_-M_ZFC_)/dT (inset in Fig. 5c) exhibits a broader peak and higher values of the ${\bar{{\rm{T}}}}_{{\rm{B}}}$ and T_B_ temperatures than those of L60 (inset in Fig. 5b) which can be correlated with a higher effective magnetic energy barrier for L40.

Moreover, the FC curves measured at 200 Oe after ZFC curves for L40 and L60 (Fig 5b,c, respectively), are similar to each other. In comparison with the FC curve of L140 (Fig. 5a), the magnetization values of L40 and L60 follow the same ferromagnetic-like behavior; however, they are noticeably higher than those of the L140. In both cases, the contribution to the FC curve coming from the SPM nanoregions does not depend on temperature; that is, when the temperature decreases below T_irr_, the monodomain nanoregions coming from an oriented state of magnetization (in contrast to random orientations on ZFC) are cooled and contribute to rise the magnetization as temperature decreases. All this was confirmed with the field-heated magnetization curves (FH), measured after the FC curves, which follow the same path of the FC data as can be seen for the L40 sample in Fig. 5c. Therefore, as was mentioned above, it is expected that under a high applied magnetic field, the LSMO thin films will exhibit FM-like behavior due to the monodomain nanoregions, when touching each other, interact strongly across their boundaries making the SPM behavior disappear when the magnetic field intensity overcomes the magnetic energy barrier associated with the anisotropic field of the SPM monodomain boundaries^30^.

The FC curves obtained when cooling the sample from 400 to 2.5 K under a high *dc* applied magnetic field of 50 kOe are illustrated in Fig. 5a,c, and they were corrected by subtracting the magnetic signal of the substrate at 50 kOe as illustrated in the Supplementary Fig. S4. Such curves correspond with to maximum magnetization reached at each temperature, and exhibit an expected FM-like behavior for all samples. However, surprising high values of magnetization are observed for the thinner L40 and L60 samples in comparison with those of the thicker L140 sample, especially in the high temperature region. This can be taken as evidence of the quality of the crystalline and magnetic structure of the L40 and L60 samples. Additionally, for L40 and L60 such high field FC curves exhibit some features that could be correlated with the transitions observed in their ZFC data. For the L40 sample, anomalies are observed at 202 K and 185 K (Fig. 5c) around the Curie temperature T_C_ = 196 K which could be associated to a second order transition as reported for the La_0.67_Sr_0.33_MnO_3_^31^. Meanwhile, the L60 sample shows a noticeable change of magnetization at 71 K and 36 K around the blocking temperature of 41 K (Fig. 5b), and barely observed around 70 K for L40 (Fig. 5c). These features can be correlated with a strong antiparallel interaction between the monodomain nanoregions by demagnetization energy barriers on their boundaries in correspondence with the wider size distribution discussed above.

On the other hand, Fig 5d,e,f show representative curves of the *in-plane* magnetization as function of the applied magnetic field, M(H) loops, for L40, L60 and L140, respectively, measured after zero field cooling at different temperatures between 2.5 K and 300 K with a maximum applied magnetic field H_MAX_ =  ± 40 kOe. The M(H) loops were corrected by subtracting the negative-slope straight line obtained from the diamagnetic response of the SiO_*x*_/Si(100) substrate as it is illustrated in the Supplementary Fig. S5, determining its slope at different temperatures as the Supplementary Fig. S6 shows for 2.5, 5 and 300 K for L40 sample. The very small substrate paramagnetic contribution (∼ 3 × 10^−3^ emu/cm^3^), here neglected, can be attributed to SiO_*x*_ layer (see the Supplementary Fig. S7).

As expected from the discussion above, all samples exhibit typical FM hysteresis loops at 2.5 K (see insets in Fig 5d,f) where the magnetization maxima M_MAX_ are in correspondence with the values of the 50 kOe-FC curves (Fig 5a,c); however, saturation is never reached in all temperature range because of the nanostructured nature of the LSMO thin films. Moreover, the initial magnetization isotherms (not illustrated here) do not display the abrupt slope change characteristic of the first-order transition, instead, they exhibit a typical continuous change of the magnetic properties associated to a second-order transition^33^.

**Figure 6:**
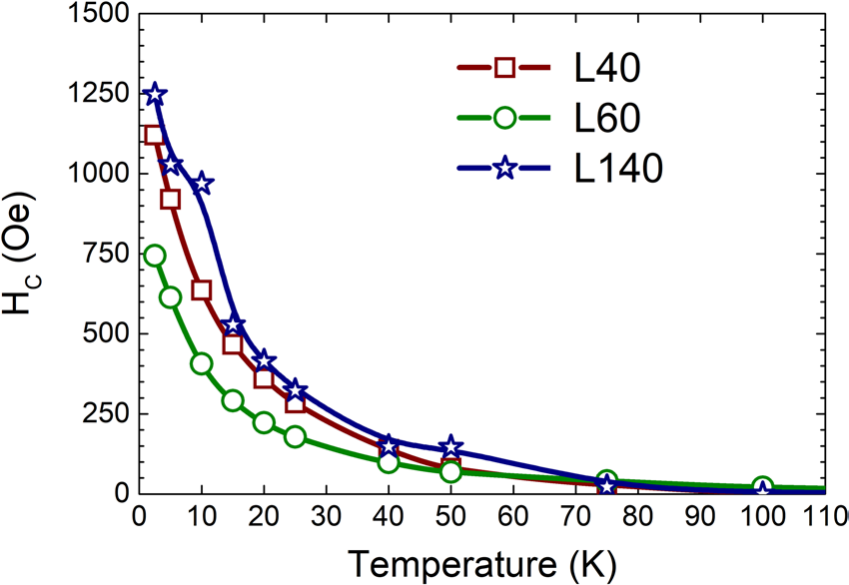
Coercive field (H_C_) values as function of temperature for the LSMO/SOS stacks.

Additionally, as can be observed in Fig 5d,f, the values of the coercive field H_C_ (illustrated in Fig. 6), the remanent magnetization and the magnetization maximum, similar for all LSMO films, decrease with the temperature increase in correspondence with the low and high applied magnetic field FC curves in Fig 5a,c. For all samples, such macroscopic ferromagnetic behavior almost disappears at temperatures above 100 K (Fig. 6). Nevertheless, surprising high values of H_C_ are observed for all samples at lower temperatures. The H_C_ values at 2.5 K of ~1250 Oe, ~746 Oe, and ~1120 Oe for L140, L60, and L40 samples, respectively, are significantly higher than those previously reported (from 10 Oe to 500 Oe) for LSMO thin films grown on different substrates such as LaAlO_3_(LAO)^20^, Ba_4_Ti_3_O_12_(BTO)/SOS and BTO/LAO/Si^11^, SrTiO_3_(STO)^11,12,20,34–37^, and STO/MgO/TiN/SOS^38^. Although the H_C_ values are significantly higher, our LSMO magnetic phase remains as a nearly soft ferromagnet exhibiting non-square loops at low temperatures. To evaluate between soft and hard behavior, the remanent-to-saturation coefficient γ = M_R_/M_S_ is usually used as a criterion^39,40^. γ ∼ 0.0 identifies a well-soft material and γ = 1 corresponds to an optimal hard material. Many reports have established the value of γ = 0.5 or 0.6 as the limit between soft and hard materials^39,40^. At 2.5 K the values of γ = M_R_(0 Oe)/ M_S_(40 kOe) are 0.35, 0.38 and 0.39 for L40, L60 and L140 samples, respectively, endorsing that our LSMO thin films are far from being hard materials.

At higher temperatures T > 100 K, the M(H) loops for all samples exhibit sigmoidal shapes (with very low values of H_C_), which can be due to weak FM ordering near the Curie transition temperature. Curves of magnetization as function of H/T measured with H_MAX_ = 40 kOe at different temperatures between 100 and 400 K for the L40 sample (with T_C_ = 196 K) are illustrated in the Supplementary Fig. S8. The anhysteretic M(H/T) curves are superimposed for temperatures above 200 K as expected from superparamagnetic behavior for temperatures above T_C_; while decreasing below 200 K the M(H/T) curves depart from the overlapped curves increasing the magnetization steeply starting from 100 K. Notice that the substrate’s magnetic contribution at high magnetic field introduces a noticeable noise in the measured data.

Furthermore, the strong interaction discussed above between the monodomain nanoregions for the thinner samples is also reflected in the M(H) loops, especially for the L60 sample characterized by its wider nanodomain size distribution. As can be seen in Fig. 5e, the shadowed hysteresis loop measured at 50 K exhibits a noticeable goose-neck shape which can be explained by the antiparallel interaction at lower applied magnetic field in the temperature region around the blocking temperature of 41 K (Fig. 5b). At higher magnetic field, the monodomains are oriented overcoming the demagnetization energy barriers and the magnetization follows a FM behavior.

## Conclusions

Highly oriented La_0.7_Sr_0.3_MnO_3_ thin films were successfully grown on SiO_x_/Si(100) substrates by rf-magnetron sputtering, with controlled thicknesses of 40, 60 and 140 nm. In contrast with previous reports on LSMO films obtained by different techniques, our results demonstrate that not buffer layer is required to obtain high quality LSMO thin films. With our approach all sputtered LSMO films were relaxed, single phase, highly dense, growing nanostructured without appreciable grain boundaries conforming locally epitaxial nanoregions with *out-of-plane* (012) preferential orientation, as a layer by layer growth of the Mn–O–Mn and La/Sr–O–La/Sr planes, induced by the constrain of the SiO_2_ surface on the native silicon oxide, with a low misorientation degree which increases with thickness.

The magnetic measurements at low applied field, represented by the M(T) curves, show a cross-over of magnetic ordering from superparamagnetic to ferromagnetic state depending of the magnetic field intensity and the LSMO thickness. The thicker film (140 nm) exhibits the typical ferromagnetic order reported for LSMO thin films but with a lower T_C_ = 218 K ascribed to its nanostructured nature. The thinner films with 40 and 60 nm exhibit a novel superparamagnetic behavior with blocking temperatures of T_B_ = 53 K and 41 K, respectively, not reported before for LSMO films, attributed to interacting ferromagnetic monodomain nanoregions with an average size smaller than the critical size required for the presence of superparamagnetism. From the M(H) hysteresis loops measurements, all LSMO films showed noticeable high coercive field values *H*_*C*_ larger than those reported for LSMO in ferromagnetic state. Our results demonstrate that sputtering it is a good fabrication technique to produce great quality magnetic nanostuctured films with high coercivity superparamagnetic behavior. As a practical conclusion, we can say that the LSMO thin films here described show potential for application as bottom electrodes for Si-based nanodevices for Spintronics.

## Supplementary information

Supplementary information.
