# Supplementary material for: Superparamagnetic state in La0.7Sr0.3MnO3 thin films obtained by rf-sputtering
Source: Sci Rep. 2020 Feb 13;10:2568. doi: 10.1038/s41598-020-59334-3 (PMC7018749; doi:10.1038/s41598-020-59334-3)
Supplement: Supplementary file 1 — Supplementary information. [file 41598_2020_59334_MOESM1_ESM.pdf]

# Supplementary Information

## Superparamagnetic state in high quality $La_{0.7}Sr_{0.3}MnO_3$ thin films obtained by rf-sputtering

M. C. Ramírez Camacho[1,2], C. F. Sánchez Valdés[3], M. Curiel[2], J. L. Sánchez Llamazares[4], J. M. Siqueiros[1], and O. Raymond Herrera[1,*].

[1]*Centro de Nanociencias y Nanotecnología, Universidad Nacional Autónoma de México, AP 14, Ensenada 22860, Baja California, México*
[2]*Instituto de Ingeniería, Universidad Autónoma de Baja California, Mexicali, Blvd. Benito Juarez s/n. 21280, Baja California, México.*
[3]*División Multidisciplinaria, Ciudad Universitaria, Universidad Autónoma de Ciudad Juárez, J. J. Macías Delgado # 18100, Ciudad Juárez 32579, Chihuahua, México.*
[4]*Instituto Potosino de Investigación Científica y Tecnológica A.C., Camino a la Presa San José 2055, Col. Lomas 4ª sección, San Luis Potosí 78216, México.*

*****Corresponding author email: raymond@cnyn.unam.mx.

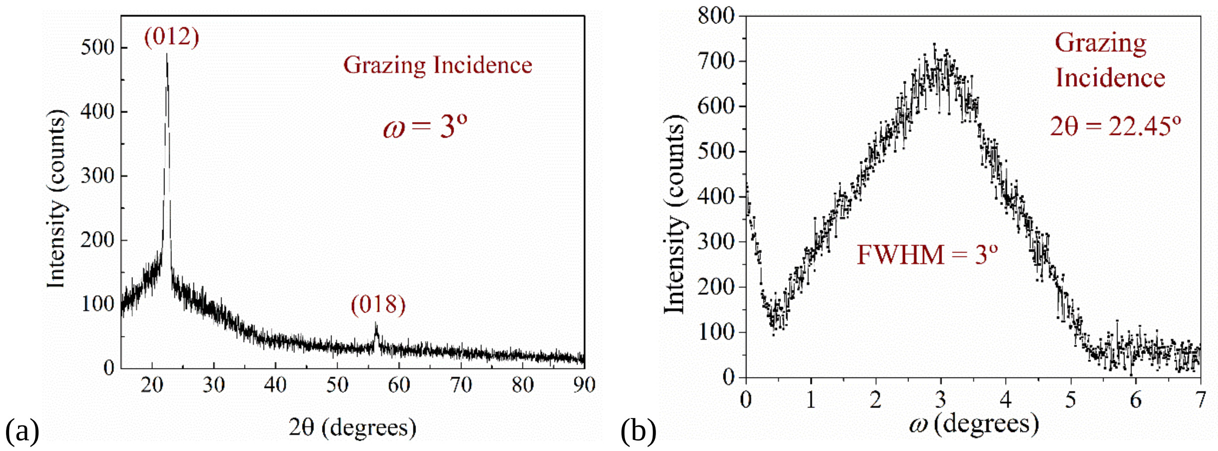


**Figure S1.** Grazing-incidence XRD profiles of the (a) omega-2theta ($\omega$-2$\theta$) scan performed at $\omega$ = 3º and (b) the $\omega$-scan (rocking curve) performed around to $\omega$ = 3º at 2$\theta$ = 22.45º in (a) corresponding to the (012) plane for the $La_{0.7}Sr_{0.3}MnO_3$ thin films with 60 nm thickness (L60 sample).

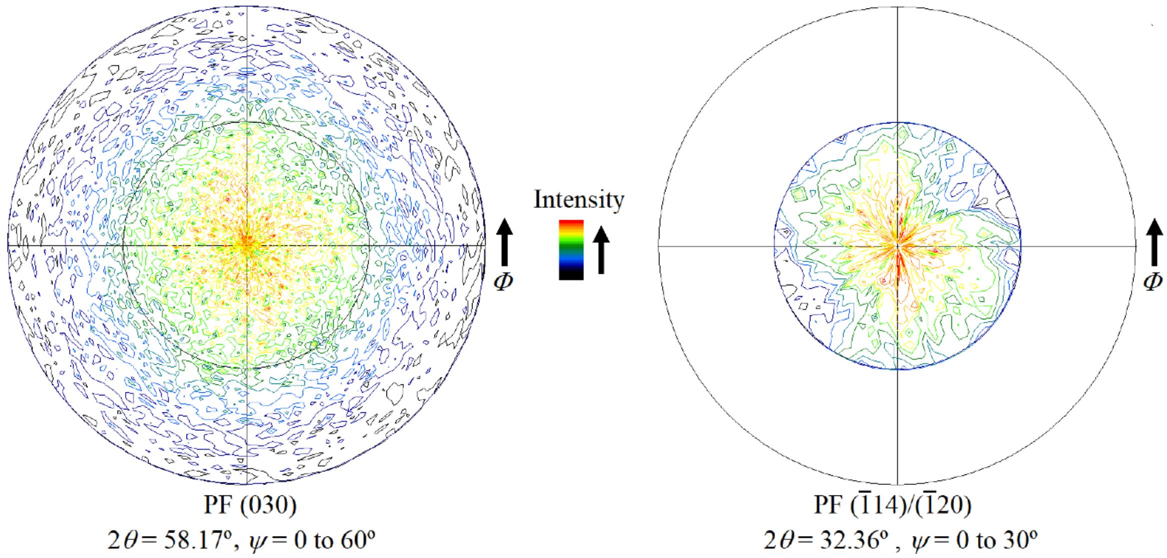


**Figure S2.** Pole figures (PF) obtained by XRD technique corresponding to (030) and $(\bar{1}14)/(\bar{1}20)$.

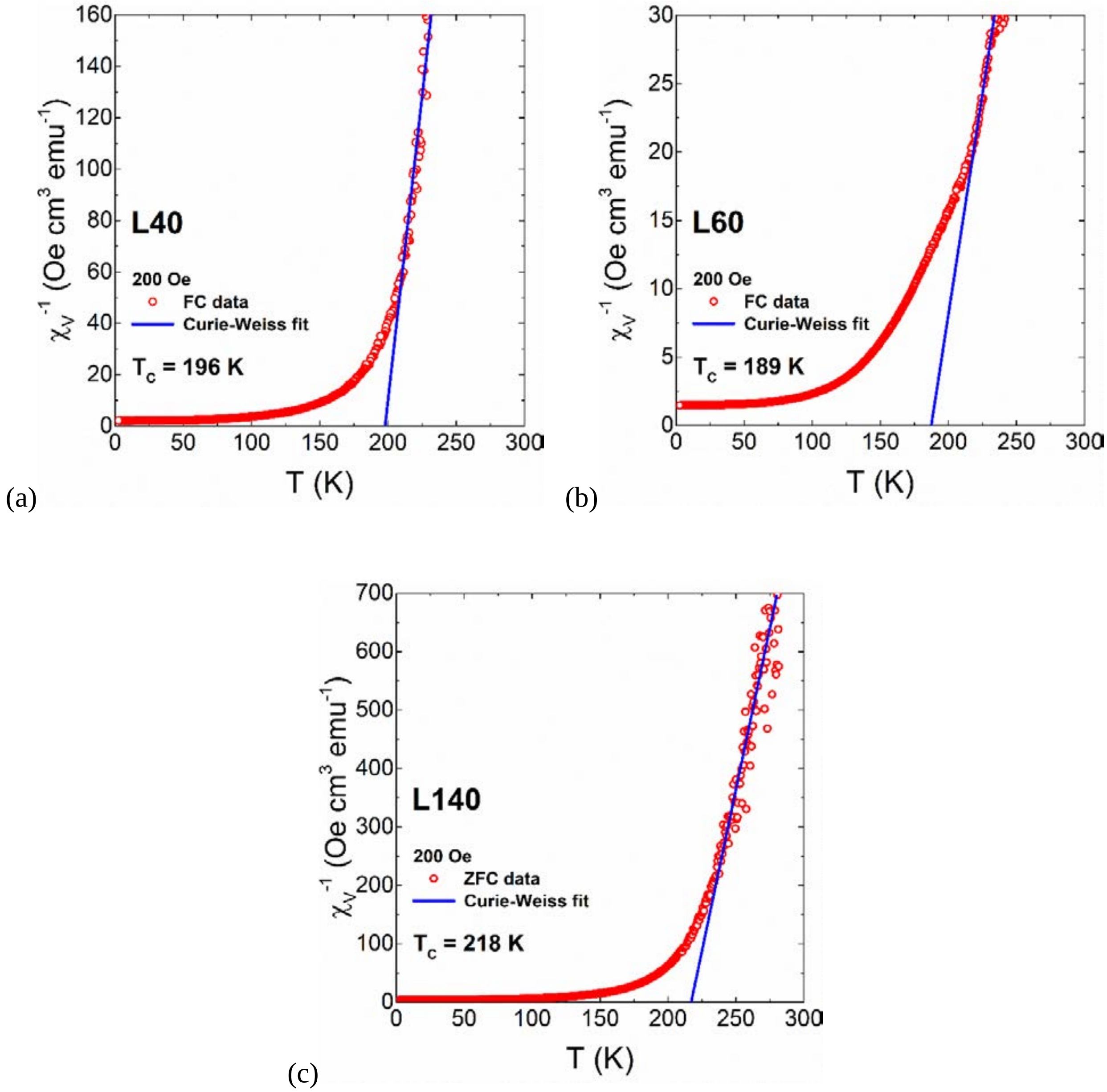


**Figure S3.** Curie temperature calculation from the reciprocal volumetric susceptibility ($\chi_V^{-1}$) plot according with the Curie-Weiss law at the high temperature region, using the field-cooled (FC) magnetization curve for the (a) L40 (40 nm) and (b) L60 (60 nm) samples exhibiting superparamagnetic behavior and using the zero-field-cooled (ZFC) magnetization curve for (c) L140 (140 nm) sample exhibiting ferromagnetic behavior.

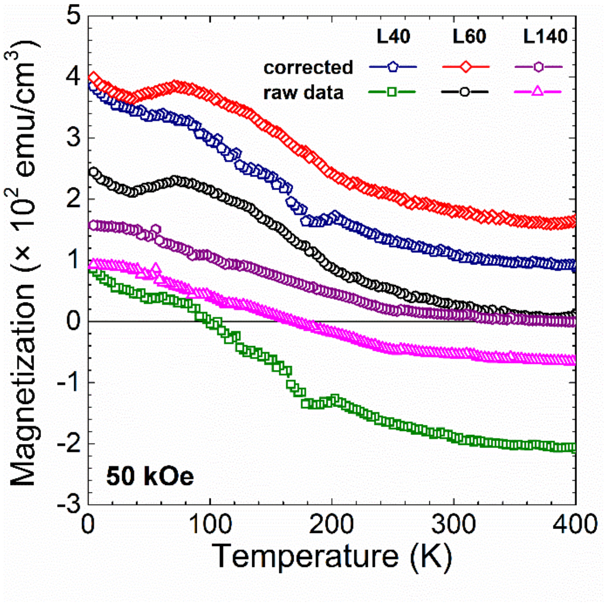


**Figure S4.** Raw and corrected data of the temperature dependence of magnetization M(T) at high magnetic field of 50 kOe for L40, L60 and L140 samples. All raw M(T) curves were corrected by subtracting the magnetic signal of the $SiO_x$/Si(100) substrate contribution.

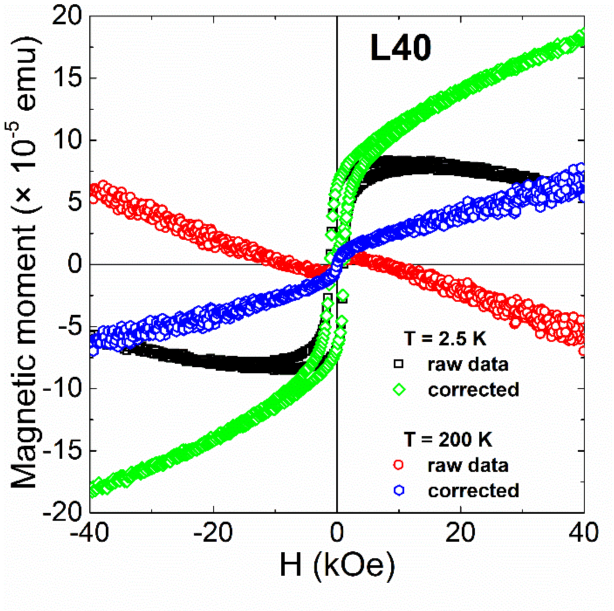


**Figure S5.** Raw and corrected data of the magnetic moment as function of the magnetic field M(H) measured up to 40 kOe at 2.5 K and 200 K for L40 sample. All raw M(H) curves were corrected by subtracting the diamagnetic contribution of the $SiO_x$/Si(100) substrate according with the Fig. S6 and S7.

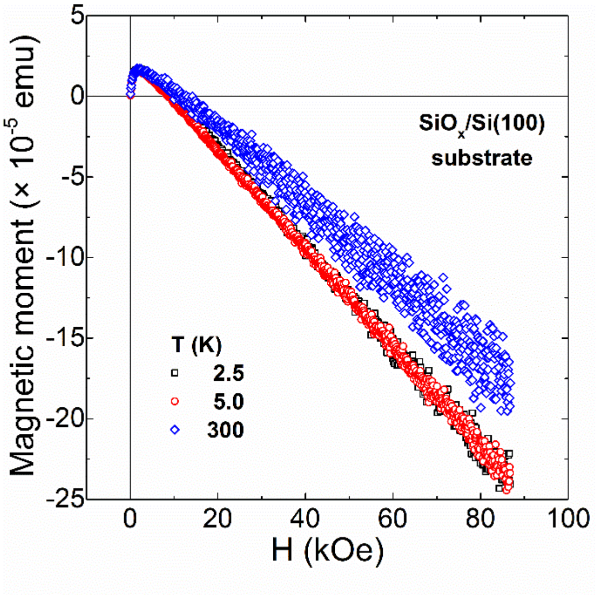


**Figure S6.** Raw data of the magnetic moment as function of the magnetic field M(H) measured up to H = 90 kOe at low (2.5 and 5 K) and room (300 K) temperatures for SiO$_x$/Si(100) substrate. The raw data illustrate the diamagnetic and paramagnetic contribution of the substrate.

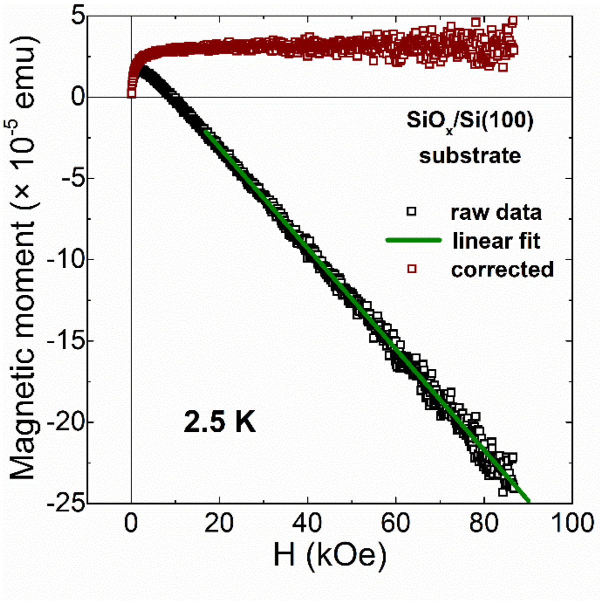


**Figure S7.** Raw and corrected data of the magnetic moment as function of the magnetic field M(H) measured up to 90 kOe at 2.5 K for SiO$_x$/Si(100) substrate. A linear fit was performed in order to correct the diamagnetic contribution of the silicon wafer.

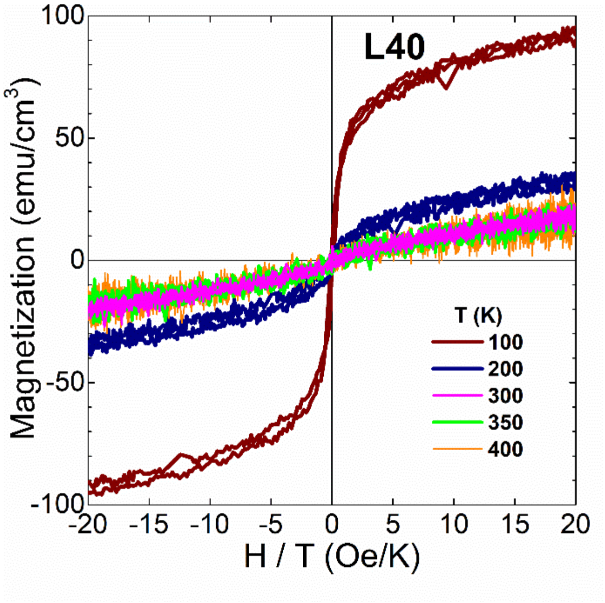


**Figure S8.** Magnetization as function of H/T (M(H/T)) curves measured with $H_{MAX}$ = 40 kOe at different temperatures between 100 and 400 K for L40 sample with $T_C$ = 196 K. The overlapping of the M(H/T) curves for temperatures above 200 K is expected from superparamagnetic behavior.
